# Editorial: *Ex-vivo* and *in-vivo* genome engineering for metabolic and neurometabolic diseases

**DOI:** 10.3389/fgeed.2023.1248904

**Published:** 2023-07-06

**Authors:** Pasqualina Colella, Vasco Meneghini, Guilherme Baldo, Natalia Gomez-Ospina

**Affiliations:** ^1^ Department of Pediatrics, Stanford University, Stanford, CA, United States; ^2^ San Raffaele Telethon Institute for Gene Therapy (SR-Tiget), IRCCS San Raffaele Scientific Institute, Milan, Italy; ^3^ Vita-Salute San Raffaele University, Milan, Italy; ^4^ Clinical Hospital of Porto Alegre, Federal University of Rio Grande do Sul, Porto Alegre, Brazil

**Keywords:** genome editing, neurometabolic, liver, retina, HSPC, metabolic, NHEJ, HDR

Recent advances in genome modification tools have led to a growing interest in using genome engineering as a therapeutic solution for many diseases. At the forefront of this revolution is the CRISPR-Cas9 technology, which made genome editing broadly accessible and engendered the development of chimeric genome editing tools like base editors and prime editors. To achieve the desired DNA modifications, nuclease-based platforms use cellular DNA repair pathways, such as Homology Directed Repair (HDR), Non-Homologous End Joining (NHEJ), and Microhomology-Mediated End Joining (MMEJ), while prime editors employ an RNA-based reverse transcription mechanism.

For therapeutic applications, genome engineering platforms can be used *ex vivo* and *in vivo* and can either disrupt coding or regulatory sequences (therapeutic NHEJ) or make precise sequence changes (therapeutic HDR, Base editing, and Prime editing). The most advanced applications of genome editing for human monogenic diseases involve therapeutic NHEJ, which uses Cas9 endonuclease and guide RNAs (gRNAs) to create site-specific double-strand breaks (DSBs), which NHEJ then repairs. This process often results in the insertion/deletion of a few nucleotides (INDELs) or larger deletions, depending on the gRNA design, mostly disrupting, or inactivating the target gene.

Therapeutic NHEJ has been successfully applied *ex vivo* to modify CD34^+^ hematopoietic stem and progenitor cells (HSPCs) from individuals affected by beta-Thalassemia (b-Thal) and Sickle cell disease (SCD), both caused by mutations in the β-globin gene (*HBB*) (Ledford, 2020; Frangoul et al., 2021). In this strategy, Cas9/gRNAs are used to reactivate the expression of the fetal γ-globin by knocking down the erythroid expression of BCL11A, its key transcriptional repressor. Data from clinical trials confirmed that γ-globin could functionally complement the deficiency of β-globin in the hemoglobin tetramers and exert an anti-sickling function. This approach can be applied to β-Thal and SCD independently from the underlying beta-globin mutations. It is also proving to be safe and effective in clinical trials, making it a likely candidate to be the first genome-editing therapy approved for human use.

In two ongoing clinical trials therapeutic NHEJ is being utilized to target Leber Congenital Amaurosis type 10 (LCA10) (Maeder et al., 2019), a form of inherited blindness, and Transthyretin Amyloidosis (ATTR), a lethal cardiomyopathy (Gillmore et al., 2021). The LCA10 trial involves the use of a single Adeno-Associated virus (AAV) vector to deliver a saCas9 and two gRNAs to delete an abnormal splice donor site in the CEP290 gene and restore CEP290 expression in the photoreceptor cells (Maeder et al., 2019). Meanwhile, the ATTR trial uses a liver-targeted nanoparticle (LNP) to deliver a Cas9 mRNA and a single gRNA to the hepatocytes *in vivo* to disrupt the expression of the TTR gene and the pathological storage of misfolded TTR protein in affected individuals (Gillmore et al., 2021). The efficacy of this approach is based on the fact that 99% of circulating TTR is secreted from the liver, and reducing its secretion would reduce the deposition of amyloid TTR in the heart (Gillmore et al., 2021).

Metabolic and neurometabolic diseases represent a significant unmet medical need and can be addressed using genome editing. However, there are several challenges that need to be overcome. Many of these conditions require targeting the central nervous system, which has proven difficult using both *in vivo* and *ex vivo* methods. Moreover, therapeutic NHEJ is unsuitable for most of these indications, and therapeutic HDR is needed instead. To shed light on these Research Topic, this Research Topic of Frontiers in Genome Editing presents a Research Topic of reviews and one research article that report and discuss the progress and obstacles of achieving efficient and safe genome editing *in vivo* in various target organs and *ex vivo* in CD34^+^ HSPCs for metabolic and neurometabolic conditions. The articles offer expert perspectives on liver (Lisjak et al.) and retinal (da Costa et al.) genome editing *in vivo*, *ex-vivo,* and *in vivo* genome editing for Friedreich’s Ataxia (Sivakumar and Cherqui), and *ex vivo* genome editing of CD34^+^ HSPCs for the treatment of non-hematological diseases (Buffa et al.).

Genetic mutations in liver-expressed genes cause many inborn errors of metabolism, and for some, organ transplantation still represents the only curative option (Fagiuoli et al., 2013). Lisjak et al. evaluated the efficiency and efficacy of CRISPR/Cas9-based genome editing at inserting a therapeutic transgene under the control of the Albumin locus in mouse hepatocytes. They used a model of hemophilia B (HB), a severe X-linked bleeding disorder caused by loss of function mutations in the coagulation factor IX (FIX) gene. Hepatic AAV-FIX gene transfer *in vivo* has successfully treated adult HB individuals, leading to the approval of the first gene therapy drug for HB (Herzog et al., 2023). However, this approach is only practical once the liver reaches adult size, as AAV genomes are lost as hepatocytes divide (Samelson-Jones and George, 2023). A similar hurdle is faced in treating severe liver metabolic diseases requiring treatment early after birth to prevent life-threatening metabolic decompensation (Kamboj, 2008). Therapeutic HDR in the liver is a promising long-term strategy for these diseases because it can correct disease-causative mutations or achieve site-specific genomic integration of the therapeutic gene in the hepatocytes (Schneller et al., 2017). Specifically, Lisjak et al. administered neonate mice with two AAVs (encoding the Cas9/gRNA and a promoterless FIX donor repair template) to stably express FIX from the Albumin locus. Integration of the FIX coding sequence in 4%–10% of hepatocytes was enough to provide stable therapeutic levels of circulating FIX up to 4–10 months after dosing and to correct HB coagulation defects in FIX-deficient mice. Low editing efficiency (0.35% of edited alleles) and subtherapeutic amounts of circulating FIX were instead reported in adult HB mice, emphasizing the inefficiency of HDR in non-dividing hepatocytes. Interestingly, increased rates of cell division in mice treated as neonates showed to favor not only HDR but also the expansion of an initial pool of edited cell clones. Achieving therapeutic levels of HDR in non-actively dividing cells still represents a challenge for many genome editing applications.

Buffa et al. Discuss the progress and challenges of *ex vivo* genome editing in HSPCs to treat non-hematological disorders. One of these challenges is achieving efficient HDR in quiescent long-term repopulating HSCs, and, at the same time, reducing the editing-associated toxicities, which may affect their long-term engraftment. Buffa et al. give an overview of HDR-dependent and -independent genome editing tools used to modify HSPCs, and existing strategies to either boost HDR or enrich the HDR-edited cell fraction. The authors also discuss the efficiency and toxicity of current methods to deliver the editing tools and HDR templates in HSPCs *ex-vivo*. Improvements in HSPC mobilization, *ex-vivo* expansion, viral and non-viral delivery methods, and recipient HSC niche conditioning are vital to developing safe and effective *ex-vivo* edited HSPC drugs. Finally, the authors review the pre-clinical studies and the current clinical applications of autologous transplantation of gene-modified HSPCs, using lentiviral vectors (LV), for the treatment of severe leukodystrophies (Cartier et al., 2009; Biffi et al., 2013) and lysosomal storage diseases (LSDs) (Gentner et al., 2021). These pivotal and successful studies and the approval of LV-modified HSPCs for the treatment of Metachromatic leukodystrophy (MLD) (Sessa et al., 2016; Fumagalli et al., 2022) and X-linked adrenoleukodystrophy (X-ALD) (Cartier et al., 2012; Eichler et al., 2017; Tucci et al., 2021) paved the way for the current pre-clinical development of autologous transplantation of genome-edited HSPCs for the treatment of LSDs. In these approaches, CD34^+^ HSPCs are engineered by the HDR-mediated insertion of therapeutic expression cassettes at safe harbor loci (e.g., CCR5) (Gomez-Ospina et al., 2019) or under the control of constitutive and cell-specific promoters to achieve supra-physiological secretion of the soluble lysosomal enzymes. Therefore, as cited by Lisjak et al. (Lisjak et al.) and Buffa et al. (Buffa et al.), therapeutic HDR to achieve site-specific integration of genes at safe arbor loci is emerging as a versatile platform to tackle recessive diseases. HDR-independent base and prime editors are potential alternatives to target mutations at the endogenous gene locus preserving physiological gene regulation.

The therapeutic benefit of HSCT for neurometabolic diseases relies on HSCT’s ability to reconstitute a healthy hematopoietic and immune system and to generate cells that engraft in visceral organs and the central nervous system (CNS) as tissue-resident macrophages (MF) and microglia-like cells (MGL) (Capotondo et al., 2012). Here, Sivakumar and Cherqui (Sivakumar and Cherqui), review the therapeutic approaches currently under investigation to treat Friedreich’s ataxia (FRDA), a disease affecting mitochondrial function and resulting in neurodegeneration, muscle degeneration, and cardiomyopathy. FRDA is caused by the deficiency of mitochondrial Frataxin (FXN), with the most common mutation consisting of the expansion of a GAA triplet repeat in *FXN* intron 1. While *in vivo* AAV-based gene therapy approaches showed promising results to restore FXN in the heart, skeletal muscle, and CNS, supraphysiological FXN is not well tolerated. *In vivo*, genome editing could also be pursued to correct FXN mutations/GAA expansion. However, the challenge of delivering the editing tools to multiple organs, including the CNS, limits this approach. If AAV vectors are used, high doses would be required to achieve the multi-systemic delivery, which still poses safety concerns. Furthermore, the expression of the genome editing tools *in vivo* raises immunogenicity concerns due to their bacterial or chimeric origin. Sivakumar and Cherqui (Sivakumar and Cherqui) describe that gene editing in HSPCs can circumvent these safety concerns and rescue the disease phenotype by excision of the FXN GAA expansion in CD34^+^ cells *ex vivo*. Upon transplantation to FRDA, the FXN-edited HSPCs are expected to cross-correct the cardiac and skeletal muscle and neurons by transferring healthy mitochondria via tunneling nanotubes.

The limited efficiency of HDR in postmitotic neurons also hampers HDR-based genome editing in retinal photoreceptors, which express causative genes of many inherited forms of blindness. HDR-independent tools, such as Base editing (BE) and Prime editing (PE), may allow us to overcome this limitation; as reviewed here by da Costa et al. (da Costa et al.), PE is the most recently developed editing tool able to potentially repair all twelve possible DNA transition and transversion mutations, as well as small insertions/deletions. However, the efficiency of PE in retinal photoreceptors has been reported to be very low (1%–2%), thus requiring further optimization. Then, due to the large size of BE and PE proteins, dual AAV vectors are needed to express them *in vivo*. Off-target indels have been observed upon delivery of BE but not PE to photoreceptors (da Costa et al.). Regardless of the approach, off- and on-target effects of genome editing need to be carefully evaluated (Hanlon et al., 2019). However, considering the immune-privileged nature of the subretinal space, the correction of photoreceptor cells *in vivo* by genome editing represents an attractive option for many inherited forms of blindness, ensuring physiological gene regulation and minimal systemic exposure to the editing tools.

In conclusion, many efforts are underway to increase the efficiency and efficacy of genome editing tools while reducing potential unintended off-target effects and toxicities. Researchers are working on ways to overcome delivery challenges for difficult targets like HSPCs, muscles, and cells in the CNS. Key factors for successful therapies for metabolic and neurometabolic disorders include optimizing non-viral delivery systems and self-inactivating editors, as well as standardizing tools for predicting and validating genotoxicity associated with genome editing. The CRISPR-Cas9 technology has propelled the field forward by offering remarkable versatility and precision. With the potential to address genetic disorders, revolutionize therapies, and enhance personalized medicine, genome editing holds immense promise for the future.
